# Comment on Onisor et al. Reply to Stein, J.M. Comment on “Onisor et al. Effectiveness and Clinical Performance of Erythritol Air-Polishing in Non-Surgical Periodontal Therapy: A Systematic Review of Randomized Clinical Trials. *Medicina* 2022, *58*, 866”

**DOI:** 10.3390/medicina61112005

**Published:** 2025-11-10

**Authors:** Holger F. R. Jentsch, Magda Mensi, Stefan-Ioan Stratul, Philipp Sahrmann, Jamal M. Stein

**Affiliations:** 1 Medical Faculty, University of Leipzig, 04109 Leipzig, Germany; 2 Section of Periodontics, School of Dentistry, Department of Surgical Specialties, Radiological Science and Public Health, University of Brescia, 25123 Brescia, Italy; magdamensi@gmail.com; 3 Department of Periodontology, Anton Sculean Center for Research and Peri-Implant Diseases, Victor Babes University of Medicine and Pharmacy, 300041 Timisoara, Romania; s.stratul@gmail.com; 4 Department of Periodontology, Endodontology and Cariology, University Center for Dental Medicine Basel UZB, CH-4058 Basel, Switzerland; philipp.sahrmann@unibas.ch; 5 Praxiszentrum für Implantologie, Parodontologie & Prothetik, 52062 Aachen, Germany; jstein@ukaachen.de; 6 Department of Operative Dentistry, Periodontology and Preventive Dentistry, University Hospital RWTH Aachen, 52074 Aachen, Germany

We comment on the reply of Onisor et al., 2025 [1] to the comment of Stein J.M., 2025 [2] regarding the systematic review of Onisor et al., 2022 [3] on the Effectiveness and Clinical Performance of Erythritol Air-Polishing in Non-Surgical Periodontal Therapy: A Systematic Review of Randomized Clinical Trials.

1. In their reply, the authors state that according to the *Cochrane Handbook for Systematic Reviews of Interventions* the use of endpoint values rather than changes from baseline is widely accepted for systematic reviews and meta-analyses. They further argue that such an approach is valid as long as baseline measurements between intervention groups are similar [1]. However, in their systematic review [3] they did not demonstrate that baseline values were statistically similar, nor was the comparability of baseline data part of their methodology. In fact, the baseline values in the studies included in their meta-analysis differed substantially, reflecting clear differences in study populations (e.g., moderate chronic periodontitis in Park et al., 2018 [4] versus stage III/IV periodontitis in Stein et al., 2021 [5] and Mensi et al., 2021 [6]). A glance at the demographic data and baseline characteristics already illustrates these differences (for example, CAL baseline values in Jentsch et al., 2020 [7]: 3.52 mm [test] vs. 3.50 mm [control]; CAL baseline values in Stein et al., 2021 [5]: 5.03 mm [test] vs. 4.99 mm [control]). These values are obviously not similar, and therefore, the effectiveness of a therapy can only be validly assessed by evaluating changes in the respective parameters, not by comparing final values alone.
2. In their reply (point 1), the authors also state that they chose to extract endpoint data as the primary outcome in order to ensure methodological consistency, comparability across studies, and to avoid selective data use. They add that this decision was made a priori, documented in their protocol, and transparently reported in the Methods section [1]. However, this cannot be recognized. On the contrary, in the Methods section (Section 2.2) of their systematic review [3], the authors explicitly defined changes in periodontal parameters as the outcome measures. Yet in the results, without any explanation or transparency, they reported endpoint values instead of the changes previously specified. This inconsistency remains unaddressed.
3. The authors further claim that, in their quality assessment, “most trials reported comparable baseline values and demonstrated low risk of bias in allocation and group comparability” and that “any minor baseline discrepancies were considered in our discussion of study heterogeneity and limitations” [1]. Again, we disagree. In the Methods section of the systematic review, the authors did not mention whether, and in particular how, comparability of baseline values was assessed [3]. Without a clear methodology for this step, the validity of their claim cannot be substantiated.
4. The authors also argue that “final values are highly relevant when assessing clinical effectiveness, particularly in reviews with heterogeneous baseline values” [1]. We respectfully disagree. The efficiency of different therapies cannot be meaningfully compared using final values without considering the actual changes achieved. A simple analogy may illustrate this: if patients with type 2 diabetes are treated with two different antidiabetic drugs, and Drug A reduces HbA1c from 9.0 to 6.5 while Drug B reduces HbA1c from 7.5 to 6.0, the approach advocated by the authors would incorrectly suggest that Drug B is more effective, simply because the final value is lower—while in fact, Drug A achieved a greater therapeutic effect. The same logic applies in the context of periodontal therapy.
5. Finally, the inconsistency in the use of the terms “effectiveness” and “efficiency” within the systematic review remains unresolved by the authors’ reply [1]. A clear distinction and consistent use of terminology are essential in scientific reporting, and this issue still requires clarification.

In conclusion, the reply of Onisor et al., 2025 [1] to the comment of Stein J.M., 2025 [2] could not resolve the methodologic problems in the systematic review of Onisor et al., 2022 [3]. The conclusions are not justified. We propose an adequate revision of the methodology including a sensitivity analysis of the included studies. Also (minor issue) the mix of “efficacy” and “efficiency” in the text stays irritating in view of the concrete objective of the study.
